# Evaluating the Timeliness of Enteric Disease Surveillance in British Columbia, Canada, 2012-13

**DOI:** 10.1155/2017/9854103

**Published:** 2017-06-01

**Authors:** Eleni Galanis, Marsha Taylor, Kamila Romanowski, Olga Bitzikos, Jennifer Jeyes, Craig Nowakowski, Jason Stone, Michelle Murti, Ana Paccagnella, Sara Forsting, Sophie Li, Linda Hoang

**Affiliations:** ^1^BC Centre for Disease Control, 655 W 12th Ave., Vancouver, BC, Canada V5Z 4R4; ^2^University of British Columbia, 2329 West Mall, Vancouver, BC, Canada V6T 1Z4; ^3^Vancouver Coastal Health, 601 W Broadway, Vancouver, BC, Canada V5Z 4C2; ^4^Interior Health, 505 Doyle Ave., Kelowna, BC, Canada V1Y 0C5; ^5^Island Health, 1952 Bay St., Victoria, BC, Canada V8R 1J8; ^6^Fraser Health, 13450-102nd Ave., Surrey, BC, Canada V3T 0H1; ^7^BC Centre for Disease Control Public Health Laboratory, 655 W 12th Ave., Vancouver, BC, Canada V5Z 4R4

## Abstract

Timely surveillance of enteric diseases is necessary to identify and control cases and outbreaks. Our objective was to evaluate the timeliness of enteric disease surveillance in British Columbia, Canada, compare these results to other settings, and recommend improvements. In 2012 and 2013, information was collected from case report forms and laboratory information systems on 2615* Salmonella*, shigatoxin-producing* E. coli*,* Shigella*, and* Listeria* infections. Twelve date variables representing the surveillance process from onset of symptoms to case interview and final laboratory results were collected, and intervals were measured. The median time from onset of symptoms to reporting subtyping results to BC epidemiologists was 26–36 days and from onset of symptoms to case interview was 12–14 days. Our findings were comparable to the international literature except for a longer time (up to 29 day difference) to reporting of PFGE results to epidemiologists in BC. Such a delay may impact our ability to identify and solve outbreaks. Several process and system changes were implemented which should improve the timeliness of enteric disease surveillance.

## 1. Introduction

Timely surveillance of enteric diseases is necessary to identify and control cases and outbreaks to prevent further transmission. Previous work conducted in British Columbia (BC), Canada, showed that 2.8 foodborne disease outbreaks per million population are reported on average every year [1] and if outbreaks are identified early, they are more likely to be solved [2]. This led us to launch a project to assess the timeliness of surveillance.

Timeliness of surveillance is defined as the time from onset of illness to the reporting of case and laboratory information to public health (PH) authorities. If this process is measured in a consistent way, we can improve our understanding of surveillance and identify steps requiring improvement. Several regions and countries have conducted this work which led to changes to improve timeliness [3–8].

In BC (population 4.6 million),* Salmonella*, shiga-toxin producing* E. coli* (STEC),* Shigella*, and* Listeria* infections are reportable to regional health authorities.* Salmonella* and STEC account for the largest number of bacterial enteric disease outbreaks in BC [1]. STEC,* Shigella*, and* Listeria* infections can cause severe acute illness and complications [9].

Our objective was to evaluate the timeliness of enteric disease surveillance in BC and to compare our results to similar work conducted in other jurisdictions internationally in order to improve the timeliness of surveillance in BC.

## 2. Methods

In BC, local laboratories report cases of* Salmonella*, STEC,* Shigella*, and* Listeria* infection to the regional health authority via fax or electronically. Cases are interviewed by regional public health officials using standard case report forms (CRF) (http://www.bccdc.ca/health-professionals/professional-resources/surveillance-forms). Although cases are reported provincially using an electronic public health information system, in 2012-13, most of the dates were kept on paper at each regional health authority.

All isolates are sent to the BC Centre for Disease Control (BCCDC) Public Health Laboratory (PHL) for further typing which includes speciation, serotyping, and pulse-field gel electrophoresis (PFGE), as required. Isolates which are difficult to serotype are sent to the National Microbiology Laboratory (NML) for serotyping. Results and dates are entered into laboratory information systems (Sunquest and Bionumerics). Partial data (confirmation, speciation, and serotyping) can be viewed by BCCDC provincial epidemiologists. All* Salmonella* Enteritidis and Heidelberg isolates are sent to the NML for phage type (PT) analysis. Results are returned by fax to the PHL and entered into a database. PFGE and PT results for* Salmonella* Enteritidis, Heidelberg, Typhimurium,* E. coli* O157:H7,* Shigella*, and* Listeria* are emailed weekly to the provincial epidemiologists for surveillance purposes.

**Figure 1:**
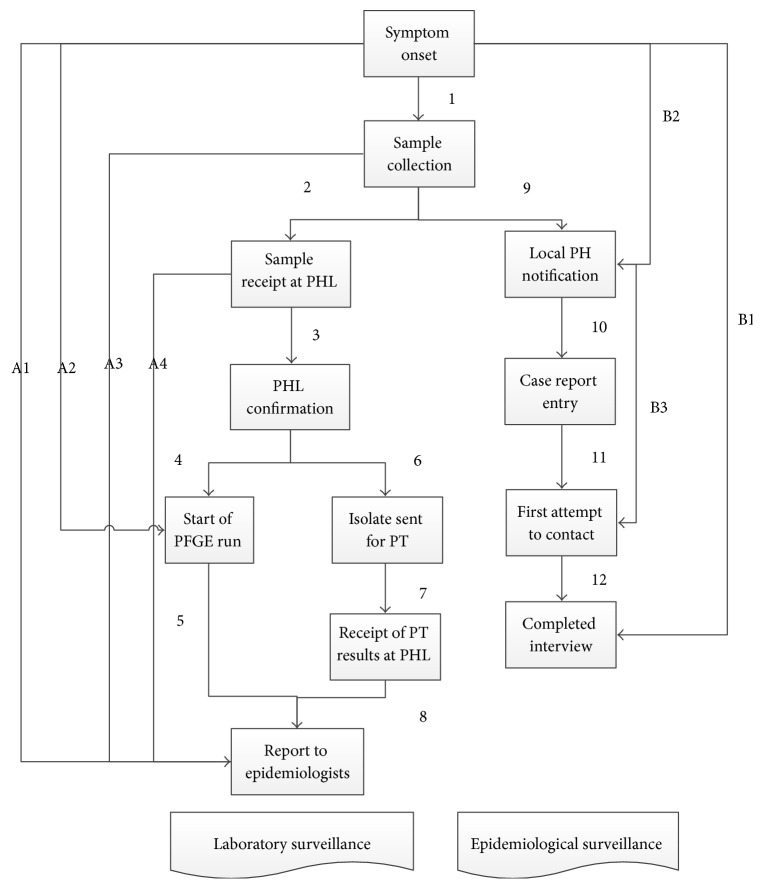
Enteric disease surveillance process and time intervals measured, British Columbia. PH: public health, PHL: public health laboratory, PFGE: pulsed-field gel electrophoresis, PT: phage type.

Date information on* Salmonella*, STEC,* Shigella*, and* Listeria* infections reported in 2012 and 2013 were collected from paper CRF and laboratory information systems. Twelve date variables were collected representing the surveillance process from onset of symptoms to case interview (epidemiological surveillance stream) and from onset of symptoms to final laboratory result provided to provincial epidemiologists (laboratory surveillance stream) (Figure 1). The data collected from paper and electronic records were linked using a unique laboratory ID and Personal Health Number. If data from different systems could not be linked due to missing or different unique identifier, only dates where intervals could be measured were included.

Time intervals between each date were measured in days. Negative intervals and intervals greater than 150 days were evaluated for accuracy. Obvious date errors were manually corrected (i.e., month and day interchanged) or excluded. Medians, ranges, and interquartile (IQ) intervals were measured for each interval and disease using MS Excel®.

Our findings were compared to similar data published from 2005–2015 identified through a literature search using Medline and through a review of the grey literature available on the Internet [3–8].

## 3. Results

Data on 2615 cases reported in 2012-13 were included:* Salmonella* (*n* = 1831), STEC (*n* = 434),* Shigella* (*n* = 323), and* Listeria* (*n* = 27). Epidemiological case data was missing for one of the five regional health authorities in 2013.

In the laboratory surveillance stream, it took a median of 3–7 days from symptom onset to sample collection and a median of 3–5 days from sample collection to sample receipt at the PHL, after initial diagnosis was completed at a local laboratory (Table 1). It took a median of 1–6 days for the PHL to issue results (including confirmatory culture where necessary and speciation or serotyping). There was a median interval of 6–8 days from PHL confirmatory culture to start the PFGE and 8–10 days from start of PFGE run to report PFGE results to provincial epidemiologists. For* Salmonella*, it took a median of 7 days to send isolates for PT, 5 days to receive the PT results, and another 9 days (IQ range = 2–22 days) to report PT results to provincial epidemiologists. The total median time from onset of symptoms to report PFGE/PT results to provincial epidemiologists was 26–36 days.

In the epidemiological surveillance stream, it took a median of 3–5 days from sample collection to the local laboratory reporting the result to the regional health authority (Table 1). The median time to enter the case report into the electronic database (0 days), to make a first attempt to interview (1 day), and to complete the interview (0 days) was negligible. However, the interval range shows that it could take up to 20 days to enter a case into the electronic database, 62 days to make a first attempt to interview a case, and 43 days to complete the interview. These ranges were highest for* Salmonella* cases. The total median time from onset of symptoms to completed interview was 12–14 days.

In general, it took more time for cases in BC to collect a sample but less time for samples to arrive and to be confirmed/serotyped at the PHL when compared to findings produced by researchers in other countries (Table 2) [3–8]. The time from sample receipt at the PHL to reporting of PFGE results to provincial epidemiologists was much longer in BC (17–33 days) than it is in some US states (4-5 days) [8]. The time from sample collection to notifying the regional health authority was a little shorter in BC, as was the time to first attempt to interview. Overall, there was a comparable interval from symptom onset to interview between BC and other jurisdictions.

## 4. Discussion

We present the first published Canadian evaluation of the timeliness of enteric disease surveillance. Understanding these time intervals has helped interpret surveillance findings (e.g., know the delay between case reporting by regional health authorities and expected lab results) and has been used during BC outbreak investigations to assess whether and when more cases or information on cases may be expected.

The longest interval was from PFGE run or PT result receipt at PHL to PFGE/PT report to provincial epidemiologists (median 8–10 days). This is in large part due to batching of PFGE and PT results which are sent to provincial epidemiologists only once a week. Individual results may be shared more rapidly during outbreak investigations. The reasons for the long interquartile range (up to 22 days) and overall ranges (up to 57 days) include repeating failed PFGE runs, awaiting NML confirmation or designation and prioritization of other laboratory work. The interval from PHL confirmation to PFGE run is about 1 week because isolates are accumulated and analysed in batches rather than immediately due to resource limitations.

The interval from onset to sample collection (3–7 days) is not highly modifiable. The longest epidemiological interval was from sample collection to notification of the regional health authority (3–5 days), which includes the diagnosis at the local laboratory; this was comparable to findings by other authors (Table 2) [4, 6].

Once the regional health authority is notified of an enteric disease, reporting and interviews are conducted quickly, on median. In BC, it is recommended that these four diseases be reported provincially and cases be contacted for interview within 24 hours of notification [10]. Our findings indicate that it can take up to 2 months for the first attempt to interview. Delays may be due to prioritization of other disease investigations or other activities, understaffing and waiting for confirmatory test results from the PHL prior to interview.

Since the epidemiological data on cases is collected much faster than the isolate subtyping data, it is important that epidemiological information be standardized and compared routinely to enable early identification of outbreaks. In BC, cases are interviewed using standard CRF and are reported regionally and provincially via electronic public health information systems. Statistical algorithms are run weekly to detect aberrations [2]. In addition, many outbreaks are identified via reporting of gastrointestinal syndromes among people who attended common events.

We found six studies which used comparable methods to measure the timeliness of surveillance in North America and Europe in the last decade [3–8]. Most of our findings were comparable to these other jurisdictions. Where they could be measured and compared, the times from symptom onset to completed interview (12–14 days in the epidemiological stream) and from symptom onset to reporting of laboratory results to epidemiologists (28 days for* Salmonella* PT in the laboratory stream) were very similar. One important difference was noted in the time from receipt of an isolate at the PHL to reporting PFGE results to provincial epidemiologists (medians of 17–33 days in BC). In a subset of US states, it takes a median of 4-5 days from isolate receipt at the state laboratory to PFGE upload into PulseNet where it is accessible by other laboratories for comparison and detection of clusters [8]. US epidemiologists in the same states also have direct real-time access to PulseNet PFGE results via SEDRIC (System for Enteric Disease Response, Investigation and Coordination) in the same 4-5 day interval (personal communications: G Biggerstaff, CDC, May 23, 2016) [11].

Although many intervals are nonmodifiable, others are. Authors agree that the most effective way to improve the timeliness of surveillance is through increasing electronic access to, or transmission of, data [12–14].

In order to improve the timeliness of enteric disease surveillance in BC, several improvements were made or planned following this study. For the laboratory surveillance stream, a pilot project will be set up to enable daily automated electronic data transmission of PFGE and PT results from the PHL to the provincial epidemiologists. In order to liberate resources to more rapidly conduct PFGE when it is most useful, the PHL has implemented an algorithm in which only the most common serotypes (e.g.,* E. coli* O157:H7) and clusters of rare serotypes (e.g., S. Montevideo) undergo routine PFGE. Epidemiologists will continue to investigate clusters of rare species and serotypes and the PHL will conduct subtyping of these isolates once a cluster is identified. Timeliness may also improve with the advent of routine whole genome sequencing of enteric pathogens, which started in 2017 in Canada. For the epidemiological stream, public health officials inform the PHL when they identify outbreaks based on epidemiological data to prioritize the testing of outbreak isolates. Finally, all five regional health authorities took measures to decrease the range of time to first attempt to interview cases. These include increased staff awareness, dedicated data entry clerks, active and timely handover of case files, and routine audits.

Although any improvements in timeliness would be helpful, it is reasonable to expect that, following our improvements, the median time from symptom onset to completed interview should remain stable at 14 days and that the median time from sample receipt at the PHL to reporting of PFGE to provincial epidemiologists should decrease by about a week to 21 days. Ultimately, improving timeliness of surveillance should improve our ability to detect and solve outbreaks. This will be measured using validated metrics such as the proportion of outbreaks solved and the time from first case to initiating an outbreak investigation [2, 15, 16].

There were some limitations to this work. Due to negative time intervals or intervals greater than 150 days, 349 dates were manually corrected and 335 dates were excluded because they could not be validated. These errors and missing data occurred randomly in all datasets and variables and would not have impacted results substantially. Overall, 29.0% of cases did not have complete data for all time periods of interest. However, since we measured the laboratory and epidemiological streams separately, this had minimal impact on the results. There were several outliers, particularly for date of onset; negative time intervals were corrected or discarded and positive outliers were kept. This may have led to a slight overestimation of intervals. Since dates from local laboratories were not available, we could not measure the time it took for local laboratories to diagnose and report cases. We were not able to identify and exclude outbreak cases; it is possible that such cases and isolates are processed faster [5].

There were several international publications on the timeliness of* Salmonella* surveillance in the last decade but few on other diseases [3, 4, 6–8]. Some of the time intervals were not directly comparable to other studies as different researchers are interested in and report on different time intervals. This is somewhat surprising given that enteric disease surveillance systems in developed countries are very similar. This may be because different jurisdictions have different diagnostic and reporting processes (e.g., different laboratories conduct different parts of the subtyping; reporting can be paper-based or electronic). Also, authors only measure or report on intervals that they are interested in or able to modify and they use different nomenclature to identify intervals. We agree with Jajosky and Groseclose that a more detailed and standardized approach, including a definition of intervals and use of the same intervals where possible, to evaluate surveillance timeliness would assist in comparability [12]. In addition, we encourage others to measure and report on the smallest intervals possible to facilitate comparison.

The US FoodCORE initiative is an attempt to set such standards at a national level [17]. Launched in 2011, 11 FoodCORE sites work together to develop improved methods to detect, investigate, and control multistate foodborne disease outbreaks. Their purposeful selection and ongoing measurement of time intervals have improved the timeliness of surveillance in participating sites [8]. Our own evaluation will be repeated after the full implementation of changes, when we also hope to see improved timeliness.

## 5. Conclusions

The measurement of the timeliness of enteric disease surveillance in BC enhanced our understanding of the surveillance process. Our findings were comparable to international benchmarks except for a considerably longer time to reporting of PFGE results. Several changes are underway to improve timeliness, including real-time electronic access to subtyping results. These will lay the foundation for other innovations such as the inclusion of genomic results and their integration with epidemiological findings to further improve our ability to identify and solve outbreaks.

## Figures and Tables

**Table 1:** Time intervals between steps in the surveillance of enteric diseases, BC, 2012-13.

	Time interval^1^	*Salmonella* median [IQ] (range) in days	STEC median [IQ] (range) in days	*Shigella *median [IQ] (range) in days	*Listeria* median [IQ] (range) in days
Laboratory surveillance	1: symptom onset to sample collection	6 [3–11] (0–163)	4 [3–8] (0–108)	7 [4–13] (0–137)	3 [1–4] (0–13)
	2: sample collection to sample receipt at PHL	5 [3–6] (0–36)	4 [2–6] (0–46)	4 [3–5] (0–20)	3 [2–4] (0–7)
	3: sample receipt at PHL to PHL confirmation (incl. serotype or species)	3 [2–4] (1–70)	6 [2–21] (1–173)	1 [1–3] (1–30)	6 [3–8] (2–69)
	4: PHL confirmation to start of PFGE run	6 [3–8] (0–55)	6 [5–10] (1–83)	8 [5–12] (0–34)	6 [5–8] (0–13)
	5: start of PFGE run to report of PFGE result to provincial epidemiologists	10 [8–22] (1–57)	8 [5–10] (1–44)	9 [7–14] (2–49)	NA^2^
	6: PHL confirmation to isolate sent for PT	7 [3–8] (0–27)	NA	NA	NA
	7: isolate sent for PT to receipt of PT result at PHL	5 [5-6] (2–26)	NA	NA	NA
	8: receipt of PT result at PHL to report of PT result to provincial epidemiologists	9 [2–22] (0–38)	NA	NA	NA
	A1: symptom onset to report of results to provincial epidemiologists	35 [25–48] (15–179)	26 [22–34] (13–99)	36 [27–49] (18–165)	NA^2^

Epidemiological surveillance	9: sample collection to regional health authority notification	4 [3–6] (0–18)	5 [3–7] (0–30)	4 [3–6] (0–35)	3 [3-4] (1–8)
	10: regional health authority notification to case report entry	0 [0-0] (0–20)	0 [0-0] (0–2)	0 [0-0] (0–3)	0 [0-0] (0-0)
	11: case report entry to first attempt to contact	1 [0–2] (0–62)	0 [0-1] (0–19)	0 [0-1] (0–31)	1 [1–5] (0–55)
	12: first attempt to contact to completed interview	0 [0-1] (0–43)	0 [0-1] (0–38)	0 [0-1] (0–29)	0 [0–3] (0–6)
	B1: symptom onset to completed interview	14 [9–21] (2–168)	12 [8–18] (3–119)	14 [10–21] (5–148)	13 [8–17] (3–61)

^1^Numbers refer to intervals in Figure 1. ^2^Dates for reporting *Listeria *PFGE to provincial epidemiologists were not available (not in regular weekly report).

**Table 2:** Comparison of time intervals between steps in the surveillance of enteric diseases between BC (2012-13) and other regions^1^.

	Time interval	*Salmonella* median in days		STEC median in days		*Shigella *median in days		*Listeria* median in days	
		BC	Int'l	BC	Int'l	BC	Int'l	BC	Int'l
Laboratory surveillance	1: symptom onset to sample collection	6	4 [3, 4]	4	3 [3]	7	2 [3]	3	NA
	2: sample collection to sample receipt at PHL	5	6 [8]	4	5 [8]	4	NA	3	6 [8]
	3: sample receipt at PHL to PHL confirmation (incl. serotype or species)	3	5 [4] 4 [8] 6 [7]	6	5 [8]	1	NA	6	NA
	A2: symptom onset to start of PFGE	21	18 [3]	19	15 [3]	22	21 [3]	NA	NA
	A3: sample collection to report of PT results to provincial epidemiologists	28	25 [4]	NA	NA	NA	NA	NA	NA
	A4: sample receipt at PHL to report of PFGE result to provincial epidemiologists	23	5 [8]	17	4 [8]	33	NA	NA	4 [8]

Epidemiological surveillance	9: sample collection to local/regional health authority notification	4	6 [4] 6 [6]	5	4 [6]	4	6 [6]	3	5 [6]
	B1: symptom onset to completed interview	14	14 [3]	12	12 [3]	14	14 [3]	13	NA
	B2: symptom onset to local/regional health authority notification	11	9 [3]	10	7 [3] 11 [5]	13	8 [3]	7	NA
	B3: local/regional health authority notification to first attempt to contact	1	2 [8]	1	2 [8]	NA	NA	4^2^	2 [8]

^1^Number in brackets refer to the associated references. ^2^Only 8 cases.
